# Investigating Irritability as a Potentially Causal Risk Pathway to Depression Using Two Genetically Informed Designs

**DOI:** 10.1016/j.bpsgos.2025.100566

**Published:** 2025-07-07

**Authors:** Amy Shakeshaft, Olakunle Oginni, Joanna Martin, Charlotte A. Dennison, Olga Eyre, Ellen Leibenluft, Sebastian Lundström, Evie Stergiakouli, Henrik Larsson, Paul Lichtenstein, Argyris Stringaris, Lucy Riglin, Mark J. Taylor, Anita Thapar

**Affiliations:** a Wolfson Centre for Young People’s Mental Health, Cardiff University, Cardiff, United Kingdom; b Centre for Neuropsychiatric Genetics and Genomics, Division of Psychological Medicine and Clinical Neuroscience, Cardiff University, Cardiff, United Kingdom; c Office of the Scientific Director, National Institute of Mental Health, Bethesda, Maryland; d Gillberg Neuropsychiatry Centre, University of Gothenburg, Gothenburg, Sweden; e MRC Integrative Epidemiology Unit, Population Health Sciences, University of Bristol, Bristol, United Kingdom; f Department of Medical Epidemiology and Biostatistics, Karolinska Institute, Stockholm, Sweden; g Faculty of Brain Sciences, Division of Psychiatry and Division of Psychology and Language Sciences, University College London, London, United Kingdom; h First Department of Psychiatry, Aiginiteion Hospital, National and Kapodistrian University of Athens, Athens, Greece

**Keywords:** Causal inference, Depression, Genetics, Irritability, Mendelian randomization, Twin study

## Abstract

**Background:**

Major depressive disorder (MDD) is heterogeneous, with diverse risk pathways leading to illness. Identifying causal routes to depression helps prioritize targets for early intervention and prevention strategies. Although irritability is associated with risk for later depression, this association could be explained by confounders, including genetic confounders.

**Methods:**

We used two genetically informed designs to examine whether irritability is causally linked to depression. First, using data from the Child and Adolescent Twin Study in Sweden (CATSS) (*N* = 16,495) and linked Swedish National Patient Register (NPR), we assessed the relationship between irritability and MDD using the monozygotic twin differences design, which controls for genetic influences. Irritability was assessed at age 15 using the Strengths and Difficulties Questionnaire. MDD diagnoses were identified from ages 15 to 25 years using the NPR. Second, we conducted bidirectional two-sample Mendelian randomization (MR) to examine relationships between genetic liability to self-reported irritability and depression, using published genome-wide association studies.

**Results:**

In CATSS, associations were observed between irritability at age 15 (parent-reported odds ratio [OR] = 1.93 [1.61–2.34], *p* = 4.65 × 10^−12^; self-reported OR = 1.62 [1.36–1.93], *p* = 7.13 × 10^−8^) and NPR-recorded MDD diagnoses from 15 to 25 years. Monozygotic twin analysis revealed an association between self-reported twin differences in irritability and MDD discordance (OR = 1.57 [1.04–2.36], *p* = .032). Results were inconclusive for parent-reported irritability (OR = 1.20 [0.73–1.96], *p* = .47). MR revealed a bidirectional relationship (irritability to depression inverse-variance weighted [IVW] OR = 3.31 [2.07–5.28], *p* = 5.5 × 10^−7^; depression to irritability IVW OR = 1.07 [1.05–1.10], *p* = 3.2 × 10^−11^).

**Conclusions:**

These results indicate that self-reported irritability may represent a causal risk pathway to MDD and thus could serve as a potential target for MDD prevention or early intervention.

## Plain Language Summary

In this study, researchers investigated whether adolescent irritability contributes causally to the development of major depressive disorder (MDD). Using two genetically informed methods, 1) twin comparisons using data from The Child and Adolescent Twin Study in Sweden and 2) Mendelian randomization, researchers found that self-reported irritability was associated with increased risk of MDD. These findings suggest that irritability may be a modifiable early risk factor for depression, highlighting its potential as a target for prevention and early intervention strategies.

It is widely acknowledged that major depressive disorder (MDD) is highly heterogeneous and that numerous risk pathways lead to the first episode of illness. An important motivation for identifying such pathways is to inform prevention and early intervention. If causal, they represent important intervention targets for preventing MDD onset, especially in high-risk groups. For example, anxiety is a well-recognized antecedent and risk pathway to later depression (1). Intervention studies have shown that early treatment of anxiety can reduce later risk of depression (2). However, anxiety is not the only pathway to MDD. A less well-researched pathway is via irritability, defined as proneness to anger that may impair an individual’s functioning (3). Multiple longitudinal studies have shown that youth irritability is associated with later depression (4, 5, 6). However, observed associations can arise from residual confounding, including genetic contributions (7). Although randomized controlled trials (RCTs) are the gold-standard design for testing causality, these are not always feasible to conduct. Quasi-experimental and genetically informed designs provide alternative approaches. However, as each design has different limitations, there is greater confidence about inferring causality when findings triangulate across multiple designs.

In this study, we draw on the strengths of two complementary genetically informed designs to examine the relationship between irritability and later MDD to infer whether there is a likely causal link. First, we use data from monozygotic (MZ) twins from a Swedish longitudinal study to control for genetic effects. Because MZ twins raised together share 100% of their genes and are assumed to have equal shared environments (8), any differences between pairs can be attributed to nonshared environmental factors, which incorporates causal processes (9, 10, 11). Here, we test whether differences in adolescent irritability symptoms between genetically identical MZ twins are associated with discordance in later MDD diagnoses. Second, we test our hypothesis using two-sample Mendelian randomization (MR). This method uses genetic variants, identified from genome-wide association studies (GWASs), as instrumental variables (IVs) for an exposure and tests for association between the IVs and the outcome to estimate the causal effect (12). MR mimics an RCT because genotypes are assigned randomly during meiosis. Together, these two complementary methods aim to triangulate evidence on the causal relationship between irritability and depression. We hypothesized that MZ differences in irritability would be associated with later MDD and that MR analyses would indicate a causal effect of genetic liability to irritability on depression.

## Methods and Materials

### Longitudinal and Twin Study Designs

#### Sample

We used data from the ongoing Child and Adolescent Twin Study in Sweden (CATSS) (13). Families of twins born in Sweden since 1992 were contacted in connection with the twins’ ninth birthday and invited to participate. Participating twins were then followed up at 15 and 18 years. A total of 41,794 individuals participated at any time in CATSS. For the current study, we used data from twins at age 15 years (total *N* = 16,495 individuals), when irritability was assessed (see Table 1 for a summary of twin zygosity). For details of study inclusion, see Figure S1. Sample sizes for each analysis are included in results tables (Table 2). All families provided written informed consent before participation, and all data were de-identified. This study received ethical approval from the Regional Ethical Review Board in Stockholm.

**Table 1:** Descriptive Statistics for the Child and Adolescent Twin Study in Sweden Sample

	Total	Female	Male
Sample Size	16,495 (100%)	8704 (52.8%)	7791 (47.2%)
Zygosity			
MZ	4870 (29.5%)	2709 (31.1%)	2161 (27.7%)
DZ, same sex	5784 (35.1%)	3016 (34.7%)	2768 (35.5%)
DZ, opposite sex	5841 (35.4%)	2979 (34.2%)	2862 (36.7%)
MDD Diagnosis, Ages 15–25	285 (1.7%)	202 (2.3%)	83 (1.1%)
Irritability Score, Parent-Report, Age 15	0.30 (0.55)	0.34 (0.58)	0.26 (0.51)
Irritability Score, Self-Report, Age 15	0.56 (0.66)	0.64 (0.68)	0.47 (0.61)

Values are presented as *n* (%) or mean (SD). MDD diagnoses and irritability scores are from the whole sample, not only MZ twins.

DZ, dizygotic; MDD, major depressive disorder; MZ, monozygotic.

#### Irritability Measures

A commonly used measure of irritability from the Strengths and Difficulties Questionnaire (SDQ) (14) was available in CATSS when twins were age 15 years: the irritability item (“often has temper tantrums”), scored 0 = “not true,” 1 = “somewhat true,” and 2 = “certainly true.” Both parent- and self-report versions were completed. This item has previously been used to investigate irritability in young people in large population-based studies (15) and has demonstrated a robust correlation (*r* = 0.49, *p* < .001) with the sum of 3 irritability items in the Development and Well-Being Assessment (DAWBA) (16,17), which is a diagnostic interview, when both measures (SDQ and DAWBA) were parent-reported (18).

#### Depression Diagnoses

CATSS is linked with the Swedish National Patient Register (NPR) (19), which contains ICD-10 codes for diagnoses from visits to specialist (secondary) inpatient and outpatient care in Sweden. Swedish clinical guidelines state that depression in individuals under 18 years should be treated in specialist child and adolescent psychiatry; therefore, the NPR should have good coverage of youth depression. Data were available from inpatient records from 1987 to December 31, 2016, and from outpatient records from 2001 to December 31, 2016. Data from primary care records were not available. Diagnoses of MDD from ages 15 to 25 years were extracted from the NPR. Because CATSS participants’ earliest year of birth is 1992, no participants have missing data at age 15 due to records not being available. Individuals who had a diagnosis of MDD prior to age 15 were excluded (*n* = 90).

#### Statistical Analysis

All statistical analyses were done using R version 4.3.3 (CATSS analysis) and version 4.2.1 (MR analysis).

First, we used the R package *drgee* to perform logistic regression analysis in all twins to assess the overall association between irritability and later MDD. Logistic regression analyses were implemented as generalized estimating equations, clustering on twin pair to control for relatedness. We carried out this analysis using parent- and self-reported irritability as predictors in separate models. Correlations between parent- and self-reported irritability were tested using Pearson correlations. Second, we derived absolute differences in parent- and self-reported irritability scores for each MZ twin pair (*n* = 2432 individual MZ twins, difference scores ranged from 0 to 2) and then categorized twin pairs into those that were discordant and concordant for MDD (coded 1 and 0, respectively). Then, we ran logistic regression analyses using irritability difference scores to predict discordance in MDD diagnosis. Again, analysis was performed using parent- and self-reported irritability in separate regression models. Because MZ twins raised together share 100% of their genetic material as well as 100% of their shared environment (8), any differences between MZ twin pairs is attributable to the nonshared environment, incorporating causal processes and measurement error (9, 10, 11).

All analyses conducted in the CATSS sample were repeated separately in males and females to test for sex differences. Because CATSS has rolling recruitment, and not all participants had reached age 25 (the upper age limit for MDD in our primary analysis), we performed additional sensitivity analyses to assess the potential impact of bias due to false negatives (i.e., participants who had not developed MDD but also not yet turned 25 by 2016, when the availability of registry data ended). To do this, phenotypic and MZ difference analyses were repeated using MDD data up to 18 years old in a subsample of the cohort who turned 18 before the end of available registry linkage (December 31, 2016). We used age 18 as the cutoff because an analysis that included only participants who had turned 25 by the end of the available registry linkage would be less well powered. The sample size using age 18 as the cutoff was 7925, of whom 170 had a diagnosis of MDD.

### Two-Sample MR

**Figure 1:**
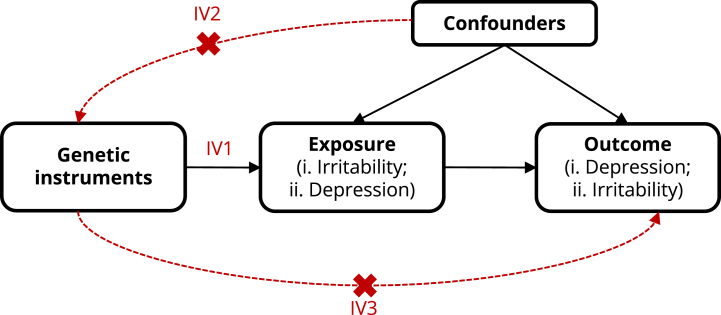
Diagram of two-sample bidirectional MR analysis between irritability and depression. MR assumptions: IV1, relevance—genetic instruments are robustly associated with the exposure; IV2, independence—genetic instruments are not associated with confounders of the exposure-outcome relationship; IV3, exclusion − restriction—genetic instruments must only be associated with the outcome via the exposure. IV, instrumental variable; MR, Mendelian randomization.

Next, we used two-sample bidirectional MR, which uses genetic variants as IVs (12), to test for causal effects between irritability and depression. The 3 core assumptions of MR are presented in Figure 1. We used summary statistics from the largest GWAS of depression available at the time of conducting analyses (166,773 cases, 507,679 controls) (20) and of self-reported irritability in the UK Biobank (UKB) from Medical Research Council Integrative Epidemiology Unit OpenGWAS (ID: UKB-b-13745) (125,001 cases, 317,168 controls) (21) to obtain independent IVs (clumping at *R*^2^ < 0.001, distance < 10,000 kb). We used *p* value < 5 × 10^−8^ as the threshold for IV selection and used *F* statistics to test for weak instrument bias (where *F* > 10 indicates sufficiently strong instruments). Because the irritability GWAS included data from the UKB, summary statistics used for depression excluded UKB data to avoid sample overlap. Both irritability and depression are measured as binary variables in their respective GWAS (see https://biobank.ctsu.ox.ac.uk/crystal/field.cgi?id=1940 for details of irritability in the UKB). Therefore, interpretation of the causal estimate from MR represents the average change in outcome per 2.72-fold increase in the prevalence of the exposure [for further details, see (22)].

MR analysis was carried out using *TwoSampleMR* (23) and *MR-PRESSO* (24) packages in R. We harmonized outcome estimates with exposure variants, so effect estimates were expressed per effect allele increase. Where effect allele frequencies were not available in exposure/outcome data, and harmonization was not possible due to being palindromic, single nucleotide polymorphisms (SNPs) were excluded from analyses. This left 43 independent IVs for irritability and 43 independent IVs for depression (see the Supplement for more detail). There was no overlap in IVs between irritability and depression. We used inverse-variance weighted (IVW) regression as the primary MR method, consistent with recommended guidance, and then examined consistency across different methods by generating estimates using weighted median, weighted mode, MR-Egger (25), MR-PRESSO [MR pleiotropy residual sum and outlier (24)], and radial MR (26). For further details of MR methods, see the Supplement. Briefly, these tests were conducted to test assumptions of MR and to assess horizontal pleiotropy, whereby IVs have an effect on the outcome through a different pathway than via the exposure (27). If these assumptions are violated, the results of MR can be biased. Thus, a consistent effect across all methods provides strong evidence for a causal effect (28). Cochran’s *Q* statistic was used to test for heterogeneity in instrument effects, whereby if *Q* > degrees of freedom, this provides evidence for heterogeneity and potentially invalid instruments. Steiger filtering and tests of directionality were also run to test directionality in the causal effect by examining the variance in exposures and outcomes explained by IVs. Where Steiger filtering indicated invalid SNPs (greater *r*^2^ for outcome than exposure), analyses were rerun using the remaining valid IVs. MR analyses are reported using Strengthening the Reporting of Observational Studies in Epidemiology (STROBE)-MR guidelines (29). For more guidance on interpretation of MR analyses, see Davies *et al.* (30).

We performed sensitivity analysis of MR results, excluding IVs for irritability which were in relative linkage disequilibrium (LD) (*r*^2^ > 0.2) with IVs for depression and vice versa. This led to excluding 4 IVs for both irritability and depression exposures (see the Supplement).

## Results

Table 1 shows descriptive statistics for the CATSS sample. Associations were observed between both parent-reported and self-reported irritability at age 15 and MDD diagnosis (Table 2). Parent-reported and self-reported irritability were moderately correlated (*r* = 0.30; 95% CI, 0.28–0.31).

**Table 2:** Results of Overall Phenotypic and MZ Difference Analyses Between Irritability and MDD in the Child and Adolescent Twin Study in Sweden Sample

Exposure	Outcome	*n*	Odds Ratio	95% CI	*p* Value
Longitudinal Analyses in the Total Sample					
Self-reported irritability	MDD	14,630 (individuals)	1.62	1.36–1.93	7.13 × 10^−8^
Parent-reported irritability	MDD	13,276 (individuals)	1.93	1.61–2.34	4.65 × 10^−12^
MZ Difference Analyses					
Self-reported irritability MZ difference	MDD MZ discordance	2094 (twin pairs)	1.57	1.04–2.36	.032
Parent-reported irritability MZ difference	MDD MZ discordance	2004 (twin pairs)	1.20	0.73–1.96	.47

MDD, major depressive disorder; MZ, monozygotic.

### MZ Discordance

Of the 2379 MZ twin pairs with complete MDD diagnosis data, 61 pairs were discordant for MDD diagnoses, 10 pairs were concordant for MDD diagnoses, and the remaining 2308 pairs were concordant for no MDD diagnosis (total *n* = 81 individuals with an MDD diagnosis, 1.7%). Associations between MZ irritability difference scores and MZ MDD discordance were observed when self-reported irritability scores were used, such that each unit increase in irritability difference was associated with a 1.57 increase in the odds of MDD discordance. However, for parent-reported irritability scores, the odds ratio was 1.20, and confidence intervals crossed the null (Table 2).

### Longitudinal and Twin Sensitivity Analyses

Sex-stratified analyses in CATSS indicated associations between both parent- and self-reported irritability and MDD in females, but in males, there was only evidence of an association for parent-reported irritability (Table S1). The sample size of concordant and discordant MZ twins was too small to meaningfully conduct separate analyses by sex. Sensitivity analysis that included only those reaching 18 years by the registry end date showed a similar pattern of results as the primary analysis (see Table S2).

### Two-Sample MR

The chance of weak instrument bias for our IVW analysis was small given instrument *F* statistics (irritability mean *F* = 37.6 [range 30–59]; depression mean *F* = 39.0 [range 28–61]). Results from bidirectional two-sample MR between irritability and depression are presented in Table 3.

**Table 3:** Results From Bidirectional Two-Sample MR of Genetic Liability to Irritability and Depression

Exposure	Outcome	No. of IVs	MR Method	Odds Ratio	95% CI	*p* Value
Irritability	Depression	43	IVW	3.31	2.07–5.28	5.5 × 10^−7^
			MR-Egger	2.36	0.16–35.84	.54
			Weighted median	1.47	0.98–2.21	.06
			Weighted mode	1.22	0.62–2.40	.57
			Radial MR	3.31	2.07–5.28	5.4 × 10^−7^
Depression	Irritability	43	IVW	1.07	1.05–1.10	3.2 × 10^−11^
			MR-Egger	1.02	0.92–1.12	.73
			Weighted median	1.06	1.04–1.08	1.4 × 10^−9^
			Weighted mode	1.06	1.00–1.12	.03
			Radial MR	1.07	1.05–1.10	2.5 × 10^−11^

IV, instrumental variable; IVW, inverse-variance weighted; MR, Mendelian randomization.

IVW estimates were consistent with a causal effect in the direction of irritability to depression (IVW OR = 3.31 [2.07–5.28], *p* = 5.5 × 10^−7^), but with evidence of heterogeneity (*Q*_41_ = 185, *p* = 3.5 × 10^−20^). See Figure S2 for MR scatterplot, forest, and leave-one-out plots. The MR-PRESSO global test indicated the presence of horizontal pleiotropy (RSS_obs_ [observed residual sum of squares] = 194, *p* < .001), and the outlier test detected 6 pleiotropic outlier SNPs (see the Supplement). When these SNPs were removed, the results remained consistent with a causal effect (OR = 2.63; 95% CI, 1.78–3.88, *p* = 1.4 × 10^−7^), and the MR-PRESSO distortion test indicated no significant change in the IVW estimate upon their removal (β = 16.7, *p* = .25). Other MR sensitivity tests (IVW radial, MR-Egger, weighted mode, and weighted median) all had effects in the same direction (OR > 1), but with wider confidence intervals, particularly for MR-Egger. The MR-Egger intercept did not indicate evidence of horizontal pleiotropy (Egger intercept = 0.0023 ± 0.0091, *p* = .81). There was evidence of regression dilution bias for MR-Egger regression (*I*^2^ = 0.24); therefore, simulation extrapolation (SIMEX) correction was applied, following Bowden *et al.* (31), although the inference remains consistent with the unadjusted MR-Egger estimates (see the Supplement). The Steiger directionality test indicated consistency with the hypothesized causal direction (irritability genetic liability to depression) (*r*^2^ exposure = 0.004, *r*^2^ outcome = 0.0004, *p* = 8.2 × 10^−108^). No IVs were removed during Steiger filtering.

MR analyses in the opposite direction (depression genetic liability to irritability) also indicated evidence of a potential causal effect, although with a small effect (IVW OR = 1.07 [1.05–1.10], *p* = 3.2 × 10^−11^). See Figure S3 for MR scatterplot, forest, and leave-one-out plots. There was evidence of heterogeneity (*Q*_41_ = 163, *p* = 3.7 × 10^−16^), and the MR-PRESSO global test detected horizontal pleiotropy (RSS_obs_ = 171, *p* < .001) and 3 pleiotropic outlier SNPs (see the Supplement). The outlier-corrected IVW estimate also indicated evidence of a causal effect (OR = 1.08; 95% CI, 1.06–1.10, *p* = 2.3 × 10^−16^), with the MR-PRESSO distortion test indicating no significant change in IVW estimate upon removal of pleiotropic outlier SNPs (β = −7.7, *p* = .50). The Steiger directionality test indicated that the causal direction was valid (*r*^2^ exposure = 0.002, *r*^2^ outcome = 0.0008, *p* = 3.5 × 10^−30^). Three IVs were removed during Steiger filtering (see the Supplement), which yielded a similar IVW estimate to the original analysis (OR = 1.06; 95% CI, 1.04–1.08, *p* = 1.65 × 10^−10^). Other MR sensitivity tests (IVW radial, MR-Egger, weighted mode, and weighted median) showed similar effects (Table 3) except for MR-Egger. The MR-Egger intercept did not indicate horizontal pleiotropy (Egger intercept = 0.0016 ± 0.0015, *p* = .29). As before, there was evidence of regression dilution bias for MR-Egger (*I*^2^ = 0.28); therefore, SIMEX correction was applied, although the inference remains consistent with the unadjusted MR-Egger estimates (see the Supplement).

### MR Sensitivity Analysis

Upon removal of IVs for irritability and depression within LD (*r*^2^ > 0.2), there was a similar pattern of results as in the primary MR analyses (Tables S3 and S4).

## Discussion

We used two complementary genetically informed designs to examine the possible causal relationship between irritability and later MDD. First, we used the MZ discordance method in a large sample of Swedish twins to test whether differences in irritability symptoms were associated with MDD when genetic contributions were controlled for. Our results showed an association between self-reported MZ twin irritability differences and MDD twin discordance but a lower effect size and confidence intervals that crossed zero for parent-reported irritability. Second, we tested our causal hypothesis using two-sample MR, which provided evidence for bidirectional causal effects between genetic liability to self-reported irritability and depression. However, the causal effect in the direction of depression genetic liability to irritability appeared small. Together, these results provide evidence that strengthens the inference of a causal relationship between self-reported irritability and depression.

Multiple longitudinal studies have shown that irritability is a risk factor for later depression, especially among youth (4,5). Although irritability and depression share genetic risk (4,32), we have shown that this relationship is independent of shared genetic influences and thus could be causal. This is consistent with evidence from a previous longitudinal twin study that examined depression symptoms rather than MDD diagnosis (6). Given our findings, it is reasonable to hypothesize that interventions targeted at reducing self-reported irritability may delay or prevent the onset of later depression, as has previously been shown for anxiety in youth (2). RCTs provide the gold standard for testing causality but are expensive and time-consuming and therefore should be based on prior evidence of a likely causal effect using quasi-experimental and genetically informative designs (7,33). The results of this study, taken together with other findings, support the rationale for conducting future treatment trials that target the reduction of self-reported irritability as a potential means of preventing depression.

An interesting and common theme in youth irritability is rater differences. In this study, results from the MZ twin design and MR were consistent with inferring a causal effect of self-reported irritability on MDD. However, MZ twin difference in parent-reported irritability was not robustly associated with MDD discordance, although we cannot rule out causal effects given our observed odds ratio. Nevertheless, this finding supplements previous evidence suggesting that irritability reported by parents and youths is not a unitary construct (34,35), a hypothesis that is supported by neuroimaging data (36). Several studies indicate that youth-reported irritability may be more reflective of emotional symptoms, which are more common during adolescence, while parent-reported irritability may be more reflective of behavioral symptoms (34,35,37), which typically onset earlier. Our results support this hypothesis and highlight the need to consider informant differences when assessing irritability in young people.

An important strength of this study is the use of two complementary methods to evaluate evidence on the causal relationship between irritability and depression, each with different strengths and limitations. The MZ twin discordance method rules out confounding by shared genetic and common environmental influences (38), and both the MZ discordance method and MR aim to overcome the challenge of associations that arise because of unmeasured confounders (7,12,39). Moreover, our MR sensitivity tests, designed to reduce the risk of pleiotropic effects, suggest that irritability is unlikely to be only a symptom or antecedent of youth depression but rather a distinct construct and developmental risk factor. Furthermore, longitudinal analyses and MR allowed us to test the direction of causality. However, we cannot be conclusive about the direction of effect because MR results indicated bidirectional effects, with a small effect in the direction of depression genetic liability to self-reported irritability.

There are also limitations of this study and the designs used. First, only one measure of irritability that includes one item (“Often has temper tantrums”) was available in CATSS. This means that only the tonic rather than the phasic aspect of irritability was examined (frequency of angry feelings/behaviors). However, this SDQ item has been used extensively to investigate irritability in young people in other large population-based studies (15) and demonstrated robust correlation (*r* = 0.49, *p* < .001) with the sum of the 3 parent-reported irritability items in the DAWBA (when SDQ was also parent-reported) (18). However, it has not been validated against self-rated DAWBA. Second, due to data availability in CATSS, we were unable to assess the impact of irritability on later MDD risk (after age 25). Also, due to sample size, our sex-stratified analysis in MZ twins was underpowered. Relating to MZ twin difference analyses, our results may underestimate the relationship between irritability and later depression, because not all young people with depression seek help or receive an MDD diagnosis from clinical services. Moreover, young adults over 18 years with depression may be under primary care, for which data are unavailable. Finally, given that CATSS participants are predominantly of European ancestry, our ability to draw conclusions about the causal link between irritability and depression in other ancestry groups is limited.

Limitations to our MR analyses are as follows: we used genetic variants associated with adult self-reported irritability and adult depression as genetic instruments, and MR provides risk effects over the whole life span. In contrast, our twin analyses included adolescents and young adults. However, polygenic scores derived from the MDD GWAS used in this study show association with adolescent MDD (40). Furthermore, there was evidence of weak instrument bias for the MR-Egger sensitivity test in both directions. Although we performed additional sensitivity checks, it is likely that regression dilution bias influences MR-Egger results. Finally, we cannot rule out the presence of pleiotropic effects in MR analyses; however, sensitivity analyses sought to address this, showing consistent results upon removal of potentially pleiotropic IVs.

### Conclusions

These novel results use two complementary genetically informed designs to strengthen the inference that irritability is a causal risk factor for depression. The findings suggest that youth irritability may be a useful target for MDD prevention and early intervention.

## Supplementary Material

Supplemental Methods, Results, Figures S1–S3, and Tables S1–S5

Key Resources Table
