# Supplementary material for: Investigating Irritability as a Potentially Causal Risk Pathway to Depression Using Two Genetically Informed Designs
Source: Biol Psychiatry Glob Open Sci. 2025 Jul 7;5(6):100566. doi: 10.1016/j.bpsgos.2025.100566 (PMC12673388; doi:10.1016/j.bpsgos.2025.100566)
Supplement: Supplemental Methods, Results, Figures S1–S3, and Tables S1–S5 [file mmc1.pdf]

SUPPLEMENTARY INFORMATION

Investigating Irritability as a Potentially Causal Risk Pathway Into Depression Using Two Genetically Informed Designs

Shakeshaft *et al.*

Supplementary text

Mendelian randomisation methods

We used inverse-variance weighted (IVW) regression as the primary MR method though estimates were also generated using weighted median, weighted mode, MR-Egger (Bowden, Davey Smith & Burgess, 2015) and MR-PRESSO (Verbanck, Chen, Neale & Do (2018)) to assess horizontal pleiotropy and MR assumptions (Slob & Burgess, 2020). Weighted median and weighted mode methods are consensus methods, unbiased by invalid IVs and robust to outliers. MR-Egger, contains an intercept term which represents the average horizontal pleiotropic effect across all IVs, and gives unbiased estimates even in the presence of horizontally pleiotropy, conditional on the assumption that the pleotropic effects are distributed independently of instrument strength (instrument strength independent of direct effect; InSIDE). MR-PRESSO is an outlier-robust method consisting of three parts, (i) the global test which detects horizontal pleiotropy, (ii) the outlier corrected causal estimate which corrects for the detected horizontal pleiotropy and (iii) the distortion test which estimates if the causal estimate is significantly different (at $p<0.05$) after adjustment for outliers. Radial MR is similar to IVW method but uses a simulation-based approach to detect outlying variants and flags them for removal in order to re-estimate the original association between the exposure and outcome (Bowden et al., 2018).

Details of instrumental variables using for MR

Irritability

There were 7977 non-independent SNPs $p<5x10^{-8}$ from the irritability GWAS (MRC IEU OpenGWAS (ID: UKB-b-13745) (Elsworth et al., 2020)). There were 46 independent SNPS left after clumping (n=340 excluded due to being missing from LD reference panel). Three SNPs were then removed during harmonization leaving 43 SNPs as IVs for analysis.

Depression

There were 3189 non-independent SNPs $p<5x10^{-8}$ from the depression GWAS (Als et al., 2023). There were 44 independent SNPs left after clumping (N=12 excluded due to being missing from LD reference panel). One SNP was then removed upon harmonization, leaving 43 SNPs to be used as instrument variables for analysis.

Removing IVs in LD

We used “ld_matrix” within TwoSample MR package to first establish LD (r^2) between SNPs used as IVs for irritability and depression. This showed 4 IVs with r^2>0.2, as shown in **Table S3**. These IVs were removed as exposures from irritability to depression MR analyses and then depression to irritability MR analyses, which were both carried out as in the primary analysis. Results from these analyses are presented in **Table S4**.

MR-Egger SIMEX adjustment

Methods

As outlined in Bowden et al. (2016), SIMEX adjustment should be used to adjust MR-Egger estimates for regression dilution, when I-squared<0.9. This adjustment was applied to the irritability to depression MR-Egger estimate using the *simex* R package, with 1000 iterations, as detailed in Bowden et al. (2016).

Results

SIMEX adjustment did not alter the inference for MR-Egger estimates, results shown **Table S5**.

MR-PRESSO outliers

The following SNPs were detected as outliers during MR-PRESSO test for irritability to depression direction: rs13157212, rs3124426, rs3935562, rs4923549, rs62055701, rs6787427. The following SNPs were detected as outliers during the MR-PRESSO test in the alternate direction (depression to irritability): rs11756123, rs2509805, rs7127383.

Steiger filtering

The following SNPS were removed during Steiger filtering of MR in the direction of depression to irritability: rs10973170, rs7127383, rs7236656.

Supplementary Tables

Table S1 - Results of phenotypic and MZ discordance analysis between irritability and MDD in CATSS, stratified by sex.

Exposure	Outcome	OR	95% CI		P-value
			Lower	Upper	
**Females**					
Self-reported irritability	MDD	1.66	1.36	2.01	4.07×10^{-7}
Parent-reported irritability	MDD	1.76	1.39	2.22	2.74×10^{-6}
Self-reported irritability MZ difference	MDD MZ discordance	1.55	0.95	2.53	0.078
Parent-reported irritability MZ difference	MDD MZ discordance	1.55	0.88	2.71	0.13
**Males**					
Self-reported irritability	MDD	1.08	0.73	1.59	0.71
Parent-reported irritability	MDD	2.15	1.56	2.96	2.36×10^{-6}
Self-reported irritability MZ difference	MDD MZ discordance	1.48	0.69	3.17	0.32
Parent-reported irritability MZ difference	MDD MZ discordance	0.54	0.17	1.73	0.30

Table S2 – Sensitivity analysis including only CATSS participants who had reached 18 years of age by the registry end date. MDD here was defined as having an onset between 15-18 years of age.

Exposure	Outcome	OR	95% CI		P-value
			Lower	Upper	
**Total**					
Self-reported irritability	MDD	1.59	1.26	2.01	8.34×10^{-5}
Parent-reported irritability	MDD	1.85	1.44	2.38	1.84×10^{-6}
Self-reported irritability MZ difference	MDD MZ discordance	2.50	1.41	4.44	0.0017
Parent-reported irritability MZ difference	MDD MZ discordance	1.35	0.73	2.52	0.34
**Females**					
Self-reported irritability	MDD	1.68	1.31	2.16	5.10×10^{-5}
Parent-reported irritability	MDD	1.69	1.23	2.31	0.0011
Self-reported irritability MZ difference	MDD MZ discordance	2.27	1.19	4.32	0.012
Parent-reported irritability MZ difference	MDD MZ discordance	1.60	0.80	3.21	0.18
**Males**					
Self-reported irritability	MDD	0.87	0.48	1.59	0.65
Parent-reported irritability	MDD	2.00	1.29	3.10	0.0019
Self-reported irritability MZ difference	MDD MZ discordance	3.00	0.85	10.51	0.09
Parent-reported irritability MZ difference	MDD MZ discordance	0.71	0.17	3.06	0.65

Table S3 – IVs removed due to LD for MR sensitivity analysis.

Irritability			Depression			R^2
SNP	Chr	Position	SNP	Chr	Position	
rs6507216	18	35195719	rs9964724	18	35159124	0.90
rs7235757	18	53067954	rs7236656	18	53107115	0.82
rs4923549	11	28637336	rs7127383	11	28591587	0.90
rs3774800	3	49334768	rs34890793	3	49619493	0.56

Table S4 – Results from MR sensitivity analysis removing irritability and depression IVs in LD.

Exposure	Outcome	N IVs	MR Method	OR	95% confidence interval		P-value
					Lower	Upper	
Irritability	Depression	39	IVW	2.43	1.61	3.66	2.1×10^{-5}
			MR Egger	5.28	0.49	56.49	0.18
			Weighted median	1.38	0.93	2.04	0.11
			Weighted mode	1.20	0.56	2.54	0.64
			Radial MR	2.43	1.62	3.66	2.0×10^{-5}
Depression	Irritability	39	IVW	1.06	1.04	1.08	1.3×10^{-9}
			MR Egger	1.03	0.94	1.13	0.54
			Weighted median	1.05	1.03	1.07	5.1×10^{-7}
			Weighted mode	1.06	1.00	1.12	0.04
			Radial MR	1.06	1.04	1.08	1.2×10^{-9}

Table S5 – Results from SIMEX adjusted and unadjusted MR-Egger tests.

Direction	Method	Beta	SE	OR	LowerCI	UpperCI	P-value
Irritability -> MDD	MR-Egger (unadjusted)	0.86	1.39	2.36	0.16	35.84	0.54
Irritability -> MDD	MR-Egger (SIMEX adjusted)	1.55	2.08	4.73	0.08	278.94	0.46
MDD -> Irritability	MR-Egger (unadjusted)	0.02	0.05	1.02	0.92	1.12	0.73
MDD -> Irritability	MR-Egger (SIMEX adjusted)	0.03	0.07	1.03	0.89	1.18	0.69

Supplementary Figures

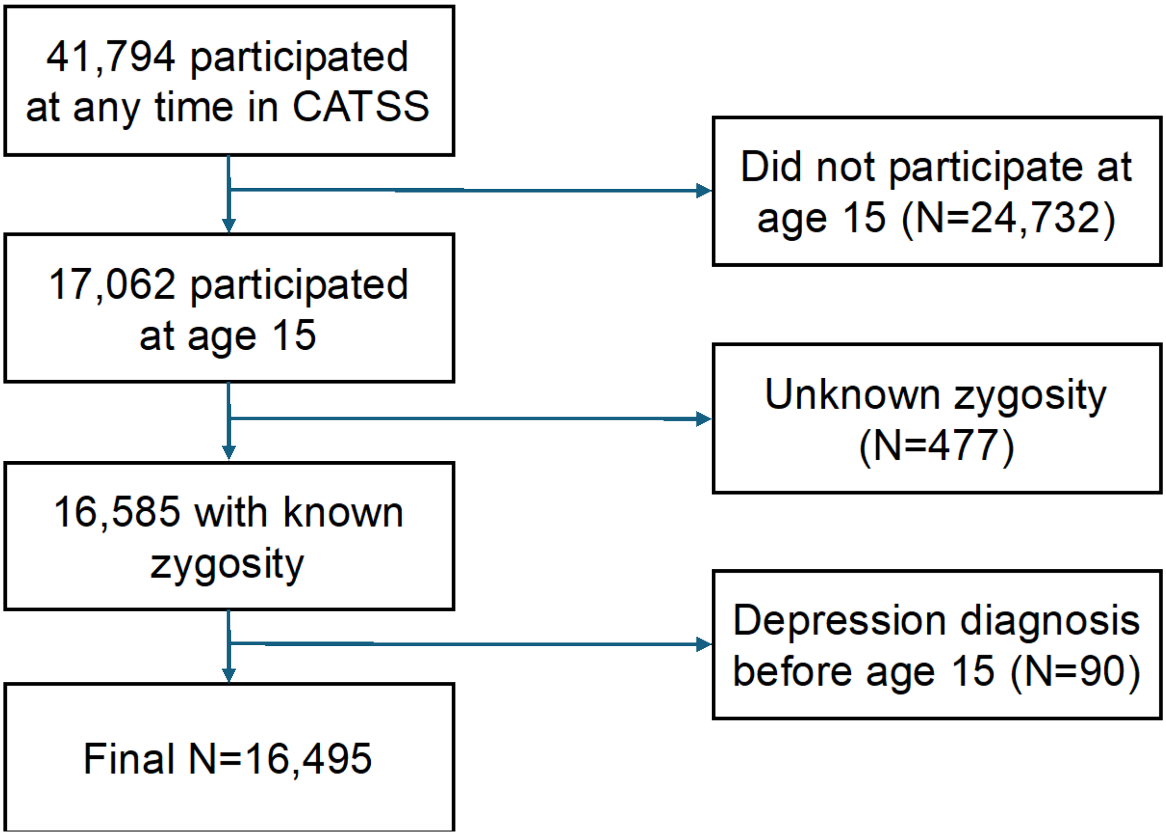

Figure S1 – Study inclusion/exclusion flowchart.

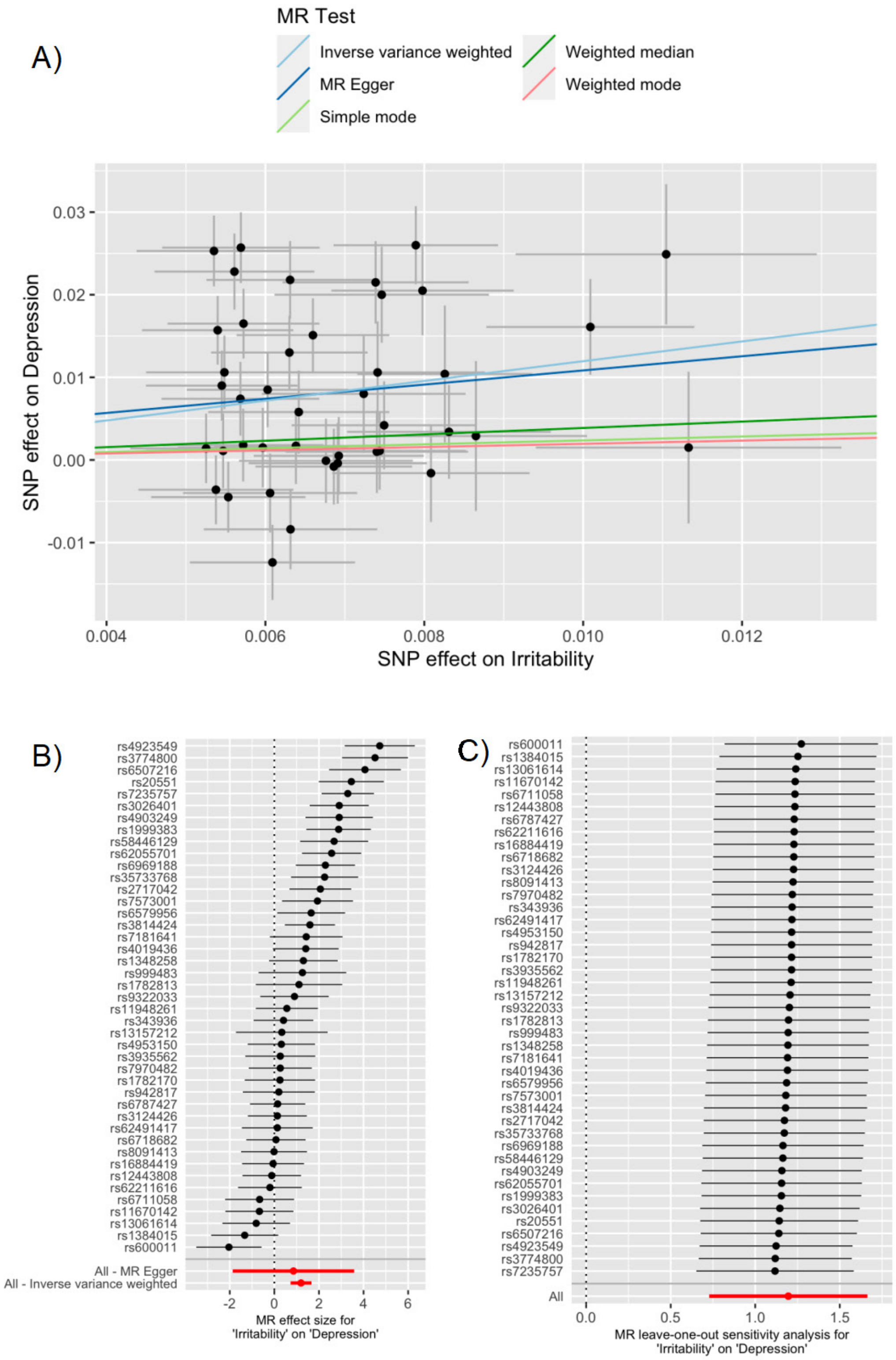

Figure S2 – Mendelian randomization (MR) plots for relationship between irritability and depression. (A) Scatter plot of single-nucleotide polymorphism (SNP) effects on irritability vs. their effects on depression. The slope of each line indicates MR effect for each method. (B) Forest plot of causal effect size of each SNP on total depression risk. (C) Leave-one-out plot for irritability to depression Mendelian randomisation (MR).

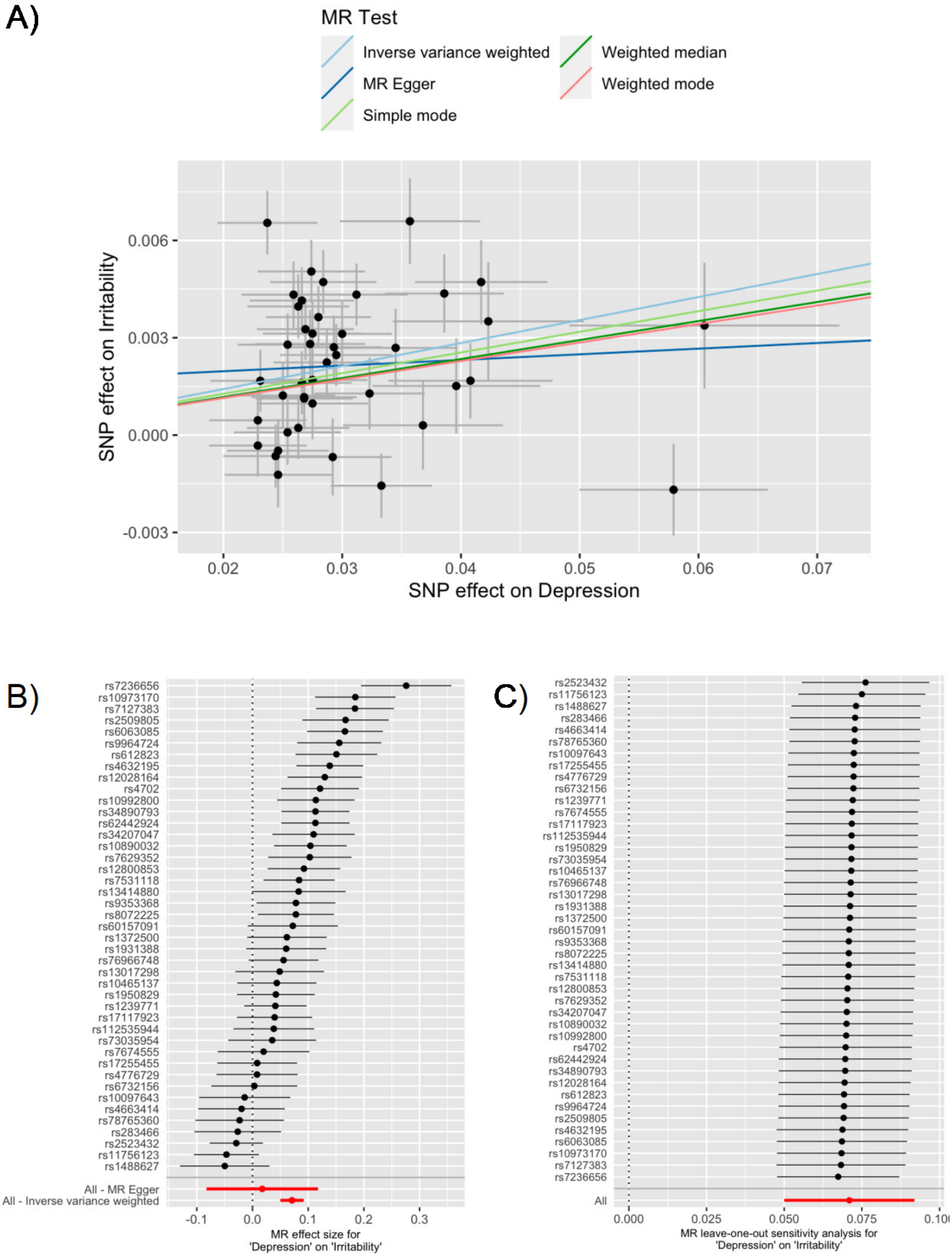

Figure S3 – Mendelian randomization (MR) plots for relationship between depression and irritability. (A) Scatter plot of single-nucleotide polymorphism (SNP) effects on depression vs. their effects on irritability. The slope of each line indicates MR effect for each method. (B)

Forest plot of causal effect size of each SNP on total irritability risk. (C) Leave-one-out plot for depression to irritability Mendelian randomisation (MR).

References

Als, T. D., Kurki, M. I., Grove, J., Voloudakis, G., Therrien, K., Tasanko, E., Nielsen, T. T., Naamanka, J., Veerapen, K., Levey, D. F., Bendl, J., Bybjerg-Grauholm, J., Zeng, B., Demontis, D., Rosengren, A., Athanasiadis, G., Baekved-Hansen, M., Qvist, P., Bragi Walters, G., Thorgeirsson, T., Stefansson, H., Musliner, K. L., Rajagopal, V. M., Farajzadeh, L., Thirstrup, J., Vilhjalmsson, B. J., Mcgrath, J. J., Mattheisen, M., Meier, S., Agerbo, E., Stefansson, K., Nordentoft, M., Werge, T., Hougaard, D. M., Mortensen, P. B., Stein, M. B., Gelernter, J., Hovatta, I., Roussos, P., Daly, M. J., Mors, O., Palotie, A. & Borglum, A. D. (2023). Depression pathophysiology, risk prediction of recurrence and comorbid psychiatric disorders using genome-wide analyses. *Nat Med,* 29**,** 1832-1844.

Bowden, J., Davey Smith, G. & Burgess, S. (2015). Mendelian randomization with invalid instruments: Effect estimation and bias detection through egger regression. *Int J Epidemiol,* 44**,** 512-525.

Bowden, J., Del Greco, M. F., Minelli, C., Davey Smith, G., Sheehan, N. A. & Thompson, J. R. (2016). Assessing the suitability of summary data for two-sample mendelian randomization analyses using mr-egger regression: The role of the i2 statistic. *Int J Epidemiol,* 45**,** 1961-1974.

Bowden, J., Spiller, W., Del Greco, M. F., Sheehan, N., Thompson, J., Minelli, C. & Davey Smith, G. (2018). Improving the visualization, interpretation and analysis of two-sample summary data mendelian randomization via the radial plot and radial regression. *Int J Epidemiol,* 47**,** 1264-1278.

Elsworth, B., Lyon, M., Alexander, T., Liu, Y., Matthews, P., Hallett, J., Bates, P., Palmer, T., Haberland, V., Smith, G. D., Zheng, J., Haycock, P., Gaunt, T. R. & Hemani, G. (2020). The mrc ieu opengwas data infrastructure. *bioRxiv***,** 2020.2008.2010.244293.

Slob, E. a. W. & Burgess, S. (2020). A comparison of robust mendelian randomization methods using summary data. *Genet Epidemiol,* 44**,** 313-329.

Verbanck, M., Chen, C. Y., Neale, B. & Do, R. (2018). Detection of widespread horizontal pleiotropy in causal relationships inferred from mendelian randomization between complex traits and diseases. *Nat Genet,* 50**,** 693-698.
